# Implementing shared decision making in federally qualified health centers, a quasi-experimental design study: the Office-Guidelines Applied to Practice (Office-GAP) program

**DOI:** 10.1186/s12913-016-1603-3

**Published:** 2016-08-02

**Authors:** Adesuwa Olomu, William Hart-Davidson, Zhehui Luo, Karen Kelly-Blake, Margaret Holmes-Rovner

**Affiliations:** 1 Department of Medicine, Michigan State University, East Lansing, USA; 2 College of Arts and Letters, Michigan State University, East Lansing, USA; 3 Department of Epidemiology and Biostatistics, Michigan State University, East Lansing, USA; 4 Center for Ethics and Humanities in the Life Sciences and Department of Medicine, Michigan State University, East Lansing, USA

**Keywords:** Shared decision-making, Patient activation, Quality improvement, Prevention of heart disease, Federally qualified health center

## Abstract

**Background:**

Use of Shared Decision-Making (SDM) and Decision Aids (DAs) has been encouraged but is not regularly implemented in primary care. The Office-Guidelines Applied to Practice (Office-GAP) intervention is an application of a previous model revised to address guidelines based care for low-income populations with diabetes and coronary heart disease (CHD). Objective: To evaluate Office-GAP Program feasibility and preliminary efficacy on medication use, patient satisfaction with physician communication and confidence in decision in low-income population with diabetes and coronary heart disease (CHD) in a Federally Qualified Healthcare Center (FQHC).

**Method:**

Ninety-five patients participated in an Office-GAP program. A quasi-experimental design study, over 6 months with 12-month follow-up. Office-GAP program integrates health literacy, communication skills education for patients and physicians, patient/physician decision support tools and SDM into routine care. Main Measures: 1) Implementation rates of planned program elements 2) Patient satisfaction with communication and confidence in decision, and 3) Medication prescription rates. We used the GEE method for hierarchical logistic models, controlling for confounding.

**Results:**

Feasibility of the Office-GAP program in the FQHC setting was established. We found significant increase in use of Aspirin/Plavix, statin and beta-blocker during follow-up compared to baseline: Aspirin OR 1.5 (95 % CI: 1.1, 2.2) at 3-months, 1.9 (1.3, 2.9) at 6-months, and 1.8 (1.2, 2.8) at 12-months. Statin OR 1.1 (1.0, 1.3) at 3-months and 1.5 (1.1, 2.2) at 12-months; beta-blocker 1.8 (1.1, 2.9) at 6-months and 12-months. Program elements were consistently used (≥ 98 % clinic attendance at training and tool used). Patient satisfaction with communication and confidence in decision increased.

**Conclusions:**

The use of Office-GAP program to teach SDM and use of DAs in real time was demonstrated to be feasible in FQHCs. It has the potential to improve satisfaction with physician communication and confidence in decisions and to improve medication use. The Office-GAP program is a brief, efficient platform for delivering patient and provider education in SDM and could serve as a model for implementing guideline based care for all chronic diseases in outpatient clinical settings. Further evaluation is needed to establish feasibility outside clinical study, reach, effectiveness and cost-effectiveness of this approach.

## Background

According to the Chronic Care Model, disease management is best provided through collaboration between the patient and the healthcare team [1]. The Patient Centered Medical Home and Meaningful Use criteria expect patients to be actively involved in the decision making and management of their chronic conditions [2–4]. Patients who become informed and active participants in decision making consistently experience a positive impact on their health outcomes [2–5]. As described by Wagner et al. high quality medical care for chronic illness must accomplish three objectives, [6]: 1) assure the delivery of those interventions that have been shown by rigorous evidence to be effective, 2) empower patients to take responsibility for the management of their condition, and 3) provide information, support, and resources to assist patients in self -management tasks. The collaborative care model described by Wagner remains an ideal but often elusive goal of care systems [6]. The use of Shared Decision Making (SDM) and decision aids (DAs) to accomplish the collaborative model has been encouraged but not regularly implemented in primary care. Shared decision-making has been defined as: “an approach where clinicians and patients share the best available evidence when faced with the task of making decisions, and where patients are supported to consider options, to achieve informed preferences” [7]. At its core, SDM rests on accepting that individual self-determination is a desirable goal and that clinicians need to support patients to achieve this goal, wherever feasible [8].

Physician and patient interventions designed in tandem to support the therapeutic partnership from both perspectives have been advocated but infrequently implemented [9]. The challenge is how to support patients and providers to develop new behaviors and knowledge about treatment options and communication skills necessary for collaboration and shared decision making (SDM). Only a few studies have simultaneously intervened with both patients and providers and objectively measure intervention effects on health outcomes [10]. Furthermore, some patient-activation interventions designed to improve patient-physician communication have been tested in patients with chronic diseases, but relatively few have used targeted strategies, and focused on ethnic minorities and low socio-economic populations who typically have lower levels of engagement and poorer communication with providers [11]. Cooper et al. [11] found that interventions that enhance physicians’ communication skills and activate patients to participate in their care positively affect patient-centered communication, patient perceptions of engagement in care and may improve systolic blood pressure among urban African-American and low socioeconomic status patients with uncontrolled hypertension. The greatest improvements were seen among patients who received coaching by a community health worker and whose physicians also received patient-centered communication skills training [11]. Stacey et al. [12] in their Cochrane review of DAs for people facing health treatment or screening decisions found high-quality evidence that DAs compared to usual care improve people’s knowledge regarding options, and reduce their decisional conflict related to feeling uninformed and unclear about their personal values. In addition, their study revealed moderately quality evidence that DAs compared to usual care stimulate people to take a more active role in decision making. They however concluded that the effects of DAs on adherence with the chosen option, cost-effectiveness, use with lower literacy populations, and level of detail needed in DAs need further evaluation [12].

Our study addresses the need for more research on use of SDM and DAs, the translation of evidence-based decision support interventions and the implementation of guidelines based care into practice especially in community outpatient settings that provide care for minority low income populations [12–15].

We previously developed an integrated shared decision making (SDM) intervention [16] based on our research in communication skills training for both patients and providers [17, 18], and provision of problem-specific decision support tools [19, 20]. Our prior study focused on decisions about exercise stress testing in stable coronary artery disease and was underpowered to evaluate the impact on patient behavior. In the present study, we used our intervention approach to encourage SDM in guidelines-based medication use in coronary heart disease (CHD) among patients with diabetes/CHD receiving care in a Federally Qualified Health Center (FQHC). FQHCs provide care for over 22 million people in 9000 communities in US [21]. They play a vital role in caring for the poor and medically underserved, underinsured, and uninsured Americans, including migrant workers and non-US citizens. Most of these patients are at higher risk of cardiovascular disease and underuse of guidelines based care [22, 23]. We tested the feasibility of the intervention process in the new application and performed a preliminary test of the impact of the intervention on patient satisfaction with communication with their provider and confidence in decision made and on medication use.

We used the approach from our previous work [16] and provided specific decision support tools to create a simple, parsimonious strategy as a new intervention in primary care we call *the Office Guidelines Applied to Practice (Office-GAP)* to improve prevention of coronary heart disease (CHD) in outpatient settings.

Our objectives were to evaluate: 1) feasibility of the Office-GAP program among patients with diabetes and CHD in a Federally Qualified Healthcare Center (FQHC); 2) the impact on a) patient satisfaction with physician communication and confidence in decisions; and b) use of guidelines-based medication for CHD prevention.

## Methods

### Design

A one-group, pretest-posttest quasi-experimental design was adopted over 6 months, followed by a 12-month follow-up. The study site was the Ingham County Healthcare Center in Mid-Michigan, a designated FQHC. Patients with diabetes and/or heart disease were recruited through direct provider referrals and on-site recruitment using patient ICD Code. At the visit to the primary practice where diabetes or heart disease, or both is on the problem list, practice staff informed patients about the study and directed interested patients to the research assistant (RA) for more information. The RA briefly described the project to the patient; if patient was interested, he/she was scheduled for a group visit; Consent and Health Insurance Portability and Accountability Act (HIPAA) forms were completed during the group visit. All providers, practice staff and patients signed informed consent documents. The Michigan State University (MSU) Institutional Review Board (IRB) approved the study.

### Inclusion criteria

Adults aged 18 or older, who could provide informed consent and who sought care from September 2009 to December 2011. ICD codes were used to identify patients with a diagnosis of 1) Diabetes mellitus. 2) Coronary heart disease. 3) All doctors and nurse practitioners providing care for patients at the study site.

### Exclusion criteria

Adults with cognitive impairment, dementia and psychosis as determined by ICD codes. Patient race was self-reported.

Interpreters were used to obtain information from patients who could not speak English. Study participants received 30-dollar reimbursement for transportation and parking fees.

All providers in the clinic and practice staff (Nurses, Medical Assistants, Administrators, Receptionists, and Social Workers) were recruited into the study. An informational meeting was offered at 4 different times to accommodate staff schedules. Two doctors (one internal medicine physician and 1 family medicine physician), 1 nurse-practitioner (NP), 8 staff, and 95 patients with a diagnosis of CAD or diabetes mellitus in one FQCHC participated in this pilot study.

### Conceptual framework

**Fig. 1:**
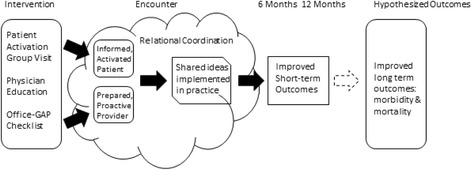
Office-GAP model

The Office-GAP model (Fig. 1) is based on the Health Literacy Care Model (HLCM) [24] and the Relational Coordination model [25]. The HLCM is a systems approach to improving patients’ engagement in care. Relational coordination refers to the quality of communication that strengthens interpersonal relationships [25, 26]. This is fundamental to collaborative goal setting that both patients and providers will embrace.

Program elements show how Office-GAP operationalizes key constructs in the Relational Coordination model that enhance relational coordination among providers and patients (Table 1). Relational coordination originally referred to the quality of communication among individuals in a work setting, and is understood as a function of the quality of those professionals’ interpersonal relationships [25]. We focused on strong provider-patient relationships as described in the HLCM [24]. The HLCM, weaves health literacy strategies into the widely accepted Wagner Care Model [6]. The Care Model represents an evidence-based framework that promotes the delivery of safe, effective, and collaborative care to patients. Measures of relational coordination have been positively correlated with outcomes in health systems [27] and integrated care in hospitals [28] and relational coordination has been shown to improve results in primary care and in community settings [29]. Havens et al. [26] report on the specific features of high-quality communication and provider relationships leading to strong relational coordination. Table 1 below summarizes the way the features of Office-GAP met the criteria discussed by Havens [26].

**Table 1:** Elements of relational coordination of shared decision making implemented in Office-GAP

High quality		
Communication is…	Office GAP Program element	Measured by
Frequent	Office visits w/GAP checklist	Use of checklist
Accurate	Decision support tools	Use of ADA/ACP^a^and Health Dialog decision Aids^b^
Problem solving	Self-management programs;	Referrals to community program^b^
	Group visit, checklist	Observation/Interview^b^
Provider-Patient		
Relationships include…		
Shared goals	Checklist	COMRADE^39^
Shared knowledge	Decision support tools	Signed copies by patient and provider
Mutual respect	Group visit checklist form	COMRADE^39^
Patient-Provider		
Relationships result in…		
Enhanced understanding of	“Here’s where we are today”	Use of checklist
Pros and cons of treatments		
Confidence in care plan	“We are on the right track”	COMRADE^39^

^a^ADA/ACP: American Diabetic Association/American College of Physician

^b^Data available upon request

*COMRADE* combined outcome measure for risk communication and treatment decision making

SDM and the HLCM both aim to improve productive interactions between informed, activated patients and a prepared, proactive practice team. The purpose is to go beyond informed decision making and paternalistic models, in which communication is one way between clinicians and patients, and to achieve two-way communication and SDM [8]. Our model explicitly identifies two aspects of the model that are often overlooked [30]. One is to directly identify the choices patients are offered (the framing problem). The framing problem in our model is answered by limiting the decision space to choices supported by guidelines. In CHD/diabetes, the clinical cases that provide the preliminary test of the model, choices offered are among known efficacious medical and behavioral treatments. The other aspect of the interaction is to deliberately address how the patient is involved (the nature of reasoning problem). The communication represented in joint decision making (the nature of reasoning) is answered by explicitly describing the pros and cons of identified treatments and encouraging the patient and physician to openly discuss each to reach a shared decision about a treatment plan. The process continues with patient values clarification and shared-decision-making to formulate a treatment plan.

### Study interventions

The Office-GAP tools were grounded in the Guidelines of the American Heart Association/American College of Cardiology (AHA/ACC) on secondary prevention of heart disease [31] and those of the American Diabetes Association (ADA) [32]. The process, developed previously [16], consisted of three Office-GAP components: 1) Physician and practice staff training; 2) Patient Group Visit; and 3) Use of the Office-GAP checklist tool during follow-up provider visits. The checklist tool [33] aided SDM and communication between patient and provider. Components were scripted and monitored to maintain study fidelity [34].

### Physician and practice staff communication skills intervention

The physician communication intervention was a 90-min training offered at 4 different times to accommodate staff schedules. Training included review of CHD secondary prevention guidelines and communication skills. Discussions on Steps in Patient-Centered Care Method of Communication (PTC) [18], SDM and goal setting, was led by Dr. Robert Smith, a world renowned expert in teaching PTC. The communication skills training goal was to increase patient engagement, activation, goal setting and empowerment. The intervention focused on strong provider-patient relationships as described in the HLCM and Relational Coordination Model. The research team, providers and practice staff discussed the best strategy for implementing the Office-GAP tools in the practice and their use during patient encounters with their providers. We identified possible opportunities and pitfalls of the study. Role-plays were conducted to model office visit skills. The training section was evaluated by surveying participating physicians and practice staff at the end of the training.

### Patient intervention

Patients attended one group visit to learn SDM, communication skills and review decision support tools parallel to skills targeted in the physician intervention. Patient pre-visit coaching has been shown to improve patients’ communication with their physicians and health outcomes [35]. We define decision support tools to include the Office-GAP checklist tool and two decision aids to support patient decision making about CHD (“Living with CHD” a 35 min DVD and Pamphlet [36] and ADA/ACP Booklet (“Living with Diabetes” [37]). How to use the GAP Agreement for Heart Health/Checklist during office visit with their physician was discussed. The Office-GAP checklist was written at the 6th grade reading level. Two follow-up visits at 3 and 6 months were scheduled with patients’ primary care physician using the Office-GAP checklist tool described in more details below. The group visits included four to six patients, were scheduled for a 90–120 min/group visit and were conducted by the Research Assistant and PI (AO).

### Group visit

Introduction to CHD and life style changes was presented through viewing the CHD decision aid [36] that included scientific information and patient interviews about secondary prevention and living with CHD. Purpose and side effects of cardiac medications were discussed by a physician (AO) with a Research Assistant. The ADA/ACP Booklet “Living with Diabetes” was reviewed to set goals. Principles of SDM and how to interact with physicians during office visit were discussed. The program focused on patient communication skills related to engagement, SDM, activation and empowerment consistent to the provider intervention skills [38, 39].

### Clinic visit

The Office-GAP Checklist (*The GAP-Agreement for Heart Health Checklist*) [33] was used to stimulate SDM and aid communication and impact the process of care by providing a systematic list of evidence based medications/ interventions for patient and provider to review together. The use of the checklist during routine office visit was an added reinforcement of the group visit SDM and patient engagement process. The Office-GAP Checklist served as the core SDM reminder tool. It was completed in *real time* by the physician and patient at two separate office visits (at 3 and 6 months). A SDM process was used to agree on medication use and life-style changes. For each guideline based item in the list, the physician checked, *Yes* (if patient was on the medication or life style activity), or *No* or *Does Not Apply to me because….* (if the patient was not eligible for the medication, had a contraindication, or was unwilling due to side effect concerns). Physician and patient discussed each item before the physician checked the box. The Office-GAP Checklist also stated the next follow-up details. At the end of the visit the physician and patient signed the checklist form to confirm that both of them has reviewed and discussed all the items. A copy of the checklist went to the medical record and a copy went to the patient to take home.

## Measures

### Intervention feasibility

The intervention feasibility was assessed by 1) Physicians’ and Office staff attendance at the providers’ educational meetings, 2) patient group visits and follow ups’ attendance, and 3) Office-GAP tool utilization rate abstracted from the medical record.

### Efficacy

1. Patient satisfaction with communication and confidence in decision
  The COMRADE survey [40] assessed the patient’s satisfaction with provider communication during the encounter, the information they received and confidence in the decisions made with their provider regarding their care. The previously validated COMRADE survey contains two sub-scales: 1) satisfaction with physician communication, and 2) patient confidence in decision made [40]. The full survey consists of 20 items that was administered at three times: at initial group visit (pre-GAP, 0 months), and at two subsequent post-GAP visits with their physician (3, 6 months) [40] It states “we would like to talk with you about your discussions with your physician” and it includes questions such as “*The doctor made me aware of the different treatments available,” “The doctor gave me a chance to be involved in the decisions during the consultation”.* “*I know the advantages of treatment or not having treatment”. “My doctor and I agreed about which treatment (or no treatment) was best for me”. “I am satisfied with the way in which the decision was made in the consultation”. “I can easily discuss my condition again with my doctor”. “I feel an informed choice has been made”.” Overall, I am satisfied with the information I was given”.*
  At baseline patients were asked to assess their satisfaction with their provider’s communication and confidence in decision made with their provider during their last office visit using the COMRADE survey.
  All items used a Likert response format (1 = strongly disagreed to 5 = strongly agree). Research Assistants explained the questions and helped only patients with limited literacy to complete the forms at each visit. Rates of using aspirin, beta-blocker, ACEI/ARBs, and cholesterol treatment were obtained at baseline, 3, 6 and 12 months as primary endpoints.
2. Medication Use
  Medication use was assessed by self-report at each visit and confirmed by patients bringing in all active medications, (validated by presence of the prescription in the medical record). This also verified that prescription was filled.

### Statistical analysis

For descriptive statistics we used mean and standard deviation (SD) for continuous variables and frequency and percentage for discrete variables. To test for the effect of the Office-GAP intervention on perceived satisfaction with communication and confidence in decision, we summed raw scores on items 1 to 10 for satisfaction subscale scores and items 11 to 20 for the confidence subscale scores. The resulting scores for both subscales could range from 10 to 50, with high scores indicating more satisfaction and more confidence. We used the generalized estimating equations (GEE) method [41] to estimate hierarchical linear models to account for the correlations of the subscale scores within the same patient, controlling for patient’s age, race, gender, primary insurance and Charlson index. These analyses tested whether the mean subscale scores were higher at each of the post Office-GAP assessment (at 3 and 6 months) than they had been at the pre-Office GAP visit (0 month/baseline).

For medication use data, we used the GEE method [41] for hierarchical logistic models, controlling for the same covariates as above. Only medication eligible patients were included in the analysis for specific adherence, resulting in different numbers of patients in each model. Medication eligibility criteria was based on guidelines and followed previous studies [42–45] (Appendix 1). “Extending our statistical analysis for medication use, we created a “global” medication adherence measure based on the following algorithm: a variable that equals to the adherence for ACEI if the patient was eligible for ACEI, else equals to the adherence for aspirin if the patient was eligible for aspirin; else equals to the adherence for statin if the patient was eligible for statin; and else equals the adherence for beta-blocker if the patient was eligible for beta-blocker.

Worst-case scenario imputation was carried out for 3 patients with missing data at 12-month follow up.

### Software

All analyses were performed using Stata 13 [46].

## Results

One hundred and forty-six patients met the inclusion criteria but only 95 (65 %) patients with CHD and/or diabetes participated in the study (Table 2). Reasons for non-participation included, refusal to participate, some indicated willingness to participate but did not show up for the group visit, some patients could not be contacted for scheduling for group visit because their phone were disconnected. On average the patients were 53.2 (SD 10.3) years of age, with BMI 36.9 (SD 10.2) and Charlson Index 2.6 (SD 1.5) indicating substantial risk of mortality within the next 10 years for a typical patient [47]. The majority of patients were female (53.7 %), white (51.6 %), with Medicare or Medicaid insurance (52.6 %), and 8.4 % were uninsured.

**Table 2:** Demographics and baseline characteristics (*N* = 95)

Continuous variable	Mean	SD
Age (years)	53.2	10.3
Body mass index (BMI, kg/m^2^)	36.9	10.2
Charlson Index*	2.6	1.5
Total No. of Office-GAP visits completed	2.4	0.8
Discrete variable	*N*	%
Female	51	53.7
Race		
White	49	51.6
African American	35	36.8
Other race/ethnicity	11	11.6
Education		
Less than high school	30	31.6
HS diploma or higher	65	68.4
Primary insurance		
Medicaid or dual	21	22.1
Medicare	29	30.5
Other	37	39.0
Uninsured	8	8.4
Smoking status		
Current smoker	38	40.0
Ex-smoker	13	13.7
Non-smoker	44	46.3
BMI Category		
< 30	21	22.1
30–35	23	24.2
> 35	51	53.7
Office-GAP visit patterns		
Patients completed only 1 visit	18	19.0
Only 2 visits	17	17.9
All 3 visits	60	63.2
Past Medical History*		
Hypertension	76	89.4
Diabetes	70	82.4
Dyslipidemia (hyperlipidemia)	65	76.5
Chronic pulmonary disease	19	22.4
Peripheral vascular disease	18	21.2
Myocardial infarction	13	15.3
Angina	10	11.8
Cerebrovascular disease	9	10.6
Congestive heart failure	8	9.4
Connective tissue disease	4	4.7
Peptic ulcer	8	9.4
Liver disease	2	2.4
Dementia	0	0
Diabetics with end organ damage	25	29.4
Renal failure	4	4.7
Any tumor	7	8.2
AIDS/Metastatic solid tumor/Leukemia/lymphoma	0	0

*% for Past medical history and Charlson index are based on 85 patients with chart review

### Intervention feasibility and program fidelity

Office-GAP was consistently implemented. All providers and staff attended the 90-min physician training. Among the 95 patients who attended the first 90-min group visits, 77 (81.1 %) completed their first Office-GAP provider visit; 60 (63.2 %) completed their second (final) visit. The Office-GAP tool was found completed in the medical record 98.7 % of the time. The clinic staff made sure that every patient goes into the consulting room with the Office-GAP checklist. We checked that both the provider and patient signed the Office-GAP checklist at the end of each encounter to confirm that both has discussed each item and they are both in agreement with their plan regarding the medication use and appropriate life-style changes. “The one-page Office-Gap checklist was reported to be simple and easy to use by the physicians in the study. The study met all the criteria for the TIDieR checklist (Appendix 2).

## Efficacy

### Communication and decision confidence

Results of the COMRADE analysis show improvement in patient satisfaction with provider communication and confidence in decisions, based on principles of SDM (Table 3). Relative to the baseline, satisfaction with communication increased at the first GAP follow-up by 4.6 points (95 % CI: 2.6, 6.5, *P* < 0.001) and by 5.0 at the second post-GAP follow-up (3.1, 7.0; *p* < 0.001). Similarly, confidence in decision increased at the first follow-up by 3.7 points (1.3, 6.1; *p* < 0.05) and by 5.5 at the second follow-up (3.0, 8.0; *p* < 0.001). These improvements were in the small to medium effect size range.

**Table 3:** Hierarchical linear models for COMRADE subscale scores

	Satisfaction *β* [95 % CI]	Confidence *β* [95 % CI]
3 months	4.55***	3.70**
	[2.63, 6.46]	[1.33, 6.07]
6 months	5.03***	5.48***
	[3.09, 6.97]	[2.96, 8.00]
Age	0.10	0.06
	[−0.04, 0.24]	[−0.09, 0.22]
Black	−1.07	−0.76
	[−4.03, 1.88]	[−4.02, 2.50]
Female	−0.61	−0.84
	[−3.37, 2.16]	[−3.90, 2.21]
Medicaid	−1.72	−1.92
	[−5.54, 2.09]	[−6.15, 2.32]
Medicare	−0.13	−0.18
	[−3.44, 3.18]	[−3.82, 3.47]
Charlson index	−1.36**	−1.18*
	[−2.34, −0.38]	[−2.25, −0.10]
Intercept	39.72***	39.32***
	[36.74, 42.70]	[35.97, 42.67]
N Obs.	205	205

Reference groups are: Pre-GAP; male; white or other race; other insurance or uninsured. Age and Charlson index are centered at the means

*COMRADE* combined outcome measure for risk communication and treatment decision making

*p < 0.05, **p < 0.01, ***p < 0.001

### Medication use

**Fig. 2:**
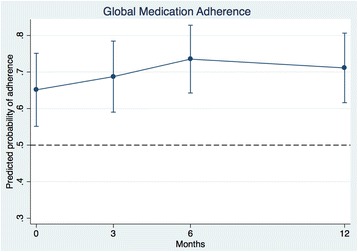
Global medication adherence

There were significant increases in the proportions of patients using Aspirin/Plavix, statin and beta-blocker during follow-up compared to baseline, with ORs for Aspirin 1.5 (95 % CI: 1.1, 2.2; *p* < 0.05) at 3-month, 1.9 (1.3, 2.9; *p* < 0.01) at 6-month, and 1.8 (1.2, 2.8; *p* < 0.01) at 12-month; for Statin 1.1 (1.0, 1.3; *p* < 0.05) at 3-months and 1.5 (1.1, 2.2; *p* < 0.05) at 12-month; and for beta-blocker 1.8 (1.1, 2.9; *p* < 0.05) at 6-month and 12-month (Table 4). The predicted probability for “global” medication adherence over time revealed that compared with baseline, the odds for adherence increased by 52 % at 6-month (OR = 1.52, 95 % CI = 1.01, 2.29; *p* < 0.05) and by 34 % at 12-month (OR = 1.34, CI = 0.87, 2.06 *p* = 0.189) (Table 4). Figure 2 depicts the predicted probability and 95 % confidence intervals for global medication adherence over time using the model in the last column in Table 4.

**Table 4:** Odds ratio [95 % CI] for medication use over time

	(1)	(2)	(3)	(4)	(5)
	Aspirin/Plavix	Statin	ACEI/ABR	Beta-blocker	“Global” medication adherence
3 months	1.50*	1.12*	1.21	1.31	1.19
	[1.05, 2.15]	[1.00, 1.25]	[0.84, 1.75]	[0.91, 1.89]	[0.85, 1.66]
6 months	1.92**	1.34	1.38	1.75*	1.52*
	[1.27, 2.92]	[0.99, 1.81]	[0.92, 2.09]	[1.07, 2.85]	[1.01, 2.29]
12 months	1.81**	1.52*	1.13	1.75*	1.34
	[1.17, 2.79]	[1.07, 2.16]	[0.72, 1.78]	[1.07, 2.85]	[0.87, 2.06]
Age	1.02	0.99	1.03	0.99	1.03
	[0.98, 1.06]	[0.94, 1.04]	[0.98, 1.08]	[0.93, 1.06]	[0.99, 1.08]
Black	0.90	1.16	0.90	0.43	0.87
	[0.36, 2.22]	[0.43, 3.08]	[0.36, 2.28]	[0.10, 1.84]	[0.35, 2.13]
Female	1.27	2.06	0.77	0.77	0.72
	[0.54, 2.97]	[0.83, 5.07]	[0.31, 1.89]	[0.19, 3.10]	[0.30, 1.70]
Medicaid	0.35	2.75	0.56	0.36	0.46
	[0.11, 1.07]	[0.80, 9.38]	[0.17, 1.81]	[0.07, 1.95]	[0.15, 1.38]
Medicare	0.96	2.16	0.71	0.70	0.74
	[0.34, 2.72]	[0.73, 6.40]	[0.24, 2.05]	[0.11, 4.68]	[0.26, 2.10]
Charlson index	1.16	1.03	0.83	1.09	0.83
	[0.85, 1.59]	[0.74, 1.42]	[0.61, 1.13]	[0.70, 1.71]	[0.61,1.12]
N obs.	300	316	296	132	332

OR with 95 % confidence intervals in brackets

Reference groups are: Pre-GAP; male; white or other race; other insurance or uninsured, Age and Charlson index centered at their respective means (53.5 and 2.7)

*p < 0.05, **p < 0.01

## Discussion

Our results demonstrate the feasibility of the Office-GAP Program and these preliminary efficacy data suggest it leads to improvement in patient satisfaction with communication with their provider and medication use. The medication use in the study was based on ACC/AHA/ADA guidelines based care for secondary prevention of heart disease for patients with diabetes and heart disease. The Office-GAP tool was found to be almost universally used in this study. The simplicity of the tools and the team-based approach which involved patients and providers/practice staff in training and implementation likely accounted for high use of Office-GAP tools. The one-page Office-GAP checklist that enables physicians’ to systematically consider evidence based care for every patient during each encounter was reported to be simple and easy to administer by the physicians. The Office-GAP decision support tools provide educational content and structure the clinical encounter.

Beyond establishing program feasibility, we are encouraged that the simultaneous training of physicians and patients in communication skills appeared to boost patients’ confidence in their decisions and their evaluation of providers’ communication as measured by COMRADE. These are critical aspects of increased trust and strengthening of the provider-patient relationship. Importantly, the improved COMRADE scores show improvement in patients’ satisfaction with provider communication and comfort with discussing prevention and treatment, and making treatment decisions with their providers. This, in itself, is an important improvement in the provider-patient relationship. In addition, the Office-GAP pilot study showed increase in medication use in this low-income population. We suggest the improved relationship may contribute to improved medication prescription use. Previous studies have rarely shown an impact of communication skills interventions on patient behavior [17]. Our results, however, are consistent with the findings of Cooper et al. [48] who showed in their Hypertension Patient-Physician Partnership Study that the greatest improvements in blood pressure control were seen among patients who received coaching by community health workers and among those whose physicians also received patient-centered communication skill training.

A key component of the system is communication skills training for both the patient and physician. A second key is that the use of the Office-GAP checklist brings the specific clinical decisions directly into the patient encounter for systematic discussion and patient-provider decision making. Results show improved physician prescribing behavior and patient follow-through. Sustained improvement in medication and life style, structured and supported by a team-based approach, could lead to improved cardiovascular outcomes [49].

We studied minority and low-income populations who are unlikely to be able to afford the cost of medications without insurance coverage. Availability of insurance coverage to almost everyone due to health care reform should sustain access to these medications.

Our feasibility study sought to implement and evaluate the proposed main intervention of a broader trial while reducing threats to the validity of the future study [50]. Our results allow estimation of the probable effects of our intervention. These results will likely enable estimation of an adequately powered sample size for a planned randomized controlled trial (RCT).

Several limitations are pertinent. Office-GAP implementation was tested in a FQHC in a small cohort and not in a randomized control trial, limiting generalizability. The educational intervention exposure for physicians was limited to a one-time administration, and may degrade over time; however, Office-GAP tools assisted the follow-up interactions. Since we did not audio or video record the encounters, there was no definitive way to confirm how physicians and patients were actually engaged in the SDM. However, at the end of the clinic visit the physician and patient signed the checklist form to confirm that both of them has reviewed and discussed all the items. In addition, we are unable to disentangle the effects of the providers’ training and the patients’ training to explain our results. The increase in medication use may reflect more effective physician prescribing and communication practices, as well as more effective patient communication and activation. We did not evaluate implementation cost in this pilot study. Finally, since we did not track which patients formed a group at each visit we could not take into account of the clustering of patients in the analysis. Despite these study limitations, this study has several strengths. We believe that the Office-GAP pilot initiative may provide the foundation for future initiatives and that it is unique in several ways. First the tools remind physicians, nurses and patients of the key goals of therapy in *real time* and in follow-up office visits. Office-GAP strengthened shared decision making and resulted in improved patient satisfaction and confidence in decision made in an underserved population not characterized by high engagement at baseline. Second, the tools’ design was very simple and easy to use at the point of care. Third, all the physicians and practice staff were involved in the training and implementation of the tools and assisted in identifying the barriers to successful implementation, a strategy previously proven to be effective in influencing physician behavior [42].

## Conclusion

The Office-GAP Program is feasible within the outpatient clinical settings. It has the potential to improve shared decision-making, satisfaction with provider communication, and evidence-based medication use for patients with heart disease and/or diabetes. Improved satisfaction with physician communication and confidence in decision may be the key to improving medication use in underserved populations. The Office-GAP Program could serve as a model for implementation of guideline based care for other chronic diseases in outpatient clinical settings. Further evaluation is needed to establish feasibility outside a clinical study, reach, effectiveness and cost-effectiveness of this approach.

## Abbreviations

CHD, Coronary Heart Disease; F/U, Follow up; NP, Nurse Practitioner; Office-GAP, Office-Guidelines Applied to Practice; PTC, Patient-Centered Method of Communication; RA, Research Assistant; SDM, Shared Decision-Making.

## Appendix 1

**Table 5:** Medication eligibility criteria. Eligibility criteria for prescribing medication to appropriate patient

Variable	Eligibility criteria
Aspirin/Plavix	Diagnosed with (1) coronary artery disease, (2) peripheral vascular disease, or (3) diabetes mellitus combined with one other risk factor (for males’ age > 50, or for females’ age > 60).
Beta-blockers	Diagnosed with (1) angina, (2) congestive heart failure paired with left ventricular systolic dysfunction, (3) coronary artery disease, (4) myocardial infarction, or (5) peripheral vascular disease.
ACEI/ARB	Diagnosed with either (1) congestive heart failure paired with an ejection fraction < 40, or (2) diabetes mellitus.
Statins & other lipid-lowering agents	Diagnosed with (1) diabetes mellitus, (2) coronary artery disease, (3) hyperlipidemia, or (4) peripheral vascular disease.

## Appendix 2

**Table 6:** TIDieR checklist. Template for intervention description and replication checklist

Item number	Item	Location	
		Primary paper	Other details
1	Brief name: Provide the name of a phrase that describes the intervention.	Page 1	Office-GAP intervention
2	Why: Describe any rationale, theory, or goal of the elements essential to the intervention	Page 6	Introduction
3	Materials: Describe any physical or informational materials used in the intervention, including those provided to participants or used in intervention delivery or in training of intervention providers. Provide information on where the materials can be accessed (e.g., online appendix, URL).	Page 12, 13	Patient and provider intervention https://www.acponline.org/practice-resources/patient-education/resources
4	Procedures: Describe each of the procedures, activities, and/or processes used in the intervention, including any enabling or support activities.	Page 13, 14	Group visit and clinic visit
5	Who Provided: For each category of intervention provider (e.g., psychologist, nursing assistant), describe their expertise, background and any specific training given.	Page 10, 14	Family Physician, Internal medicine Physician, Nurse Practitioner, Research Assistant
6	How: Describe the modes of delivery (e.g., face-to-face or by some other mechanism, such as internet or telephone) of the intervention and whether it was provided individually or in a group.	Page 13, 14	Face-to-face group visit
7	Where: Describe the type(s) of location(s) where the intervention occurred, including any necessary infrastructure or relevant features.	Page 9	Group visit and clinic
8	When and How Much: Describe the number of times the intervention was delivered and over what period of time including the number of sessions, their schedule, and their duration, intensity or dose.	Page 13	Patient intervention, group visits, and 2 follow up visits
9	Tailoring: If the intervention was planned to be personalized, titrated or adapted, then describe what, why, when, and how.	N/A	Intervention was not personalized, titrated, or adapted
10	Modifications: If the intervention was modified during the course of the study, describe the changes (what, why, when, and how).	N/A	
11	Planned: If intervention adherence or fidelity was assessed, describe how and by whom, and if any strategies were used to maintain or improve fidelity, describe them.	Page 14, 15	Intervention feasibility
12	Actual: If intervention adherence or fidelity was assessed, describe the extent to which the intervention was delivered as planned.	Page 17, 18	Intervention feasibility and program fidelity
